# TIGIT Signaling Pathway Regulates Natural Killer Cell Function in Chronic Hepatitis B Virus Infection

**DOI:** 10.3389/fmed.2021.816474

**Published:** 2022-02-21

**Authors:** Juan Wang, Hongyan Hou, Lie Mao, Feng Wang, Jing Yu, Ying Luo, Qun Lin, Ziyong Sun

**Affiliations:** ^1^Department of Blood Transfusion, Tongji Medical College, Tongji Hospital, Huazhong University of Science and Technology, Wuhan, China; ^2^Department of Laboratory Medicine, Tongji Medical College, Tongji Hospital, Huazhong University of Science and Technology, Wuhan, China

**Keywords:** hepatitis B virus (HBV), chronic hepatitis B, natural killer (NK) cell, TIGIT, cytokine secretion, cytotoxicity

## Abstract

**Background and Objective:**

Persistent infection of hepatitis B virus (HBV) and liver damage in immune active chronic hepatitis B (CHB) could be partly due to the overreaction of natural killer (NK) cells, including pro-inflammatory cytokine secretion and cytotoxicity. An immunosuppressive receptor, T-cell immunoglobulin and immunoreceptor tyrosine–based inhibitory motif (ITIM) domain (TIGIT) is specifically expressed in NK cells. This study aims to investigate the role of the TIGIT signaling pathway in regulating NK cell functions in patients with CHB.

**Method:**

We comparatively assessed the expression of TIGIT in NK cells of patients with immune active CHB (CHB-IA), carriers of immune control chronic HBV (CHB-IC), and healthy controls (HCs), and then explored mechanisms of the TIGIT signaling pathway in regulating NK cell-mediated liver injury by different molecular assessments.

**Result:**

The expression of TIGIT in NK cells was enhanced in CHB-IC but was reduced in CHB-IA compared with the HC group. In patients with CHB-IA, the expression of TIGIT was inversely correlated with intensity of the liver damage. Moreover, TIGIT-NK cells show higher IFN-γ secretion capability, degranulation activity, and cytotoxicity but lower apoptosis than TIGIT+ NK cells. Blockade of the TIGIT pathway with anti-TIGIT antibody increased NK cell function, while activation of the TIGIT pathway with TIGIT Fc and CD155 Fc chimera protein down-regulated NK cell function.

**Conclusion:**

Our data showed that the TIGIT signaling pathway participates in NK cell impairment, which could be used as a new therapeutic target to protect patients with chronic HBV infection from severe liver injury.

## Introduction

Among the primary reasons for liver diseases, chronic hepatitis B virus (HBV) infection has an estimated 350 million carriers globally (1). It has been reported that 14–30% of patients with chronic hepatitis B (CHB) suffer from cirrhosis with end-stage liver disease and hepatocellular carcinoma (HCC) (2), which causes 786,000 deaths per year (3). Normally, chronic infection of HBV involves immune tolerant (IT), immune activated (IA), immune control (IC), and immune escape phases (4). The IA phase is associated with rapid disease progression and recurrent necroinflammation, while the IC phase (previously called the inactive carrier phase) is often associated with lowered risk of HCC and cirrhosis (1, 4). However, differences in immune mechanisms between patients with CHB-IA and those with CHB-IC are largely unknown, and exploring the mechanisms of why patients with CHB-IA suffer from serious liver injuries and complications might provide a new therapeutic target for early diagnosis and appropriate treatment.

Persistent infection of HBV, death of hepatocytes, and liver injury might be due to the dysfunction of immune system. The liver of patients with CHB with damaged liver cells usually have high levels of non-viral specific lymphocytes. Natural killer (NK) cells make up 30–50% of intrahepatic lymphocytes and play vital roles against HBV infection (5, 6), but uncontrolled NK cell activation in an infected tissues may also lead to chronic immunopathology in these tissues (7). Previous studies have demonstrated that the cytotoxicity and cytokine production of NK cells correlate with the extent of damage to the liver in murine hepatitis models and patients with CHB (8, 9). Some studies have reported that during the CHB-IA stage, liver injury could lead to increased NK cell cytotoxicity (9, 10), which was caused by high levels of interleukins (ILs)-12,−15, and−18 *in situ* and low level of IL-10 (10). Moreover, hepatocyte necrosis (11), mediated by TRAIL, NKG2D/NKG2D ligand, and Fas/Fas-ligand, is another important factor (12). Main findings of the current literature on the activation of NK cells over CHB progression are reduced cytotoxicity, NKp30 and NKG2D expression, and production of IFN-γ (13). However, findings of studies on the activation and functioning of NK cells and their regulating mechanisms are controversial that necessitate conducting further studies to investigate the roles and activating mechanisms of NK cell functions in HBV infection.

T-cell immunoglobulin and immunoreceptor tyrosine–based inhibitory motif (ITIM) domain (TIGIT) is a newly found inhibitory molecule (14), which is expressed in NK cells and T cells at high levels. The TIGIT signaling pathway has been reported to be involved in the progression of various tumors (15–19), autoimmune diseases (20, 21), and chronic infections (15, 22, 23). Previous studies have shown that TIGIT was highly expressed on NK cells from murine acute viral hepatitis and negatively regulated NK-cell activation (24), which reduced liver injury and facilitated liver regeneration (25). Moreover, treatment of HCV infection results in downregulated TIGIT expression in T cells in the case of untreated severe infection (26). In viral hepatitis, TIGIT limits the activity of NK cells *via* the ITIM domain. However, the role of the TIGIT pathway in NK cell activity regulation in patients with CHB is yet to be explored.

This study aimed to evaluate the role of TIGIT in regulating NK cell function in patients with CHB. We found that the expression of TIGIT in NK cells decreased in CHB-IA compared with CHB-IC and HCs, and that TIGIT-NK cells show higher IFN-γ secretion capability, degranulation activity, and cytotoxicity but lower apoptosis than TIGIT+ NK cells. Furthermore, TIGIT signaling pathway blockade could restore the activity of NK cells, and activation of this pathway exerted opposite effect on NK cell function. Overall, we discovered a mechanism, for the first time, of TIGIT-mediated regulation of NK cell function, which may act as a novel target for protection of patients with CHB from acute inflammation and liver injury.

## Materials and Methods

### Subjects of This Study

Experimental procedures of this study were approved by the local ethics committee of Tongji Hospital, Tongji Medical College, Huazhong University of Science and Technology, Wuhan, China (ethic code: TJ-IRB20210225), and were in accordance with the ethical standards and regulations of human studies of the Helsinki Declaration (27). Study population included patients with CHB-IA (*n* = 74), CHB-IC (*n* = 40), and healthy controls (HCs) (*n* = 40) who were referred to the Tongji Hospital, Wuhan, China. The age of the participants was between 18 and 60 years old, and they did not undergo immunosuppressive drugs or antiviral therapy within 6 months prior to sampling. Each participant provided written informed consent and was required to fill in a questionnaire regarding his or her medical history and associated treatments.

Each patient fulfilled the amended criteria for CHB (4, 28), patients with CHB-IA were HBsAg+ and HBeAg+ for more than 6 months and ALT > ULN. They were also detected with fluctuating or high levels of HBV replication (>2,000 IU/ml of HBV DNA levels) (4). CHB-IC (previously called inactive carrier) were HBsAg+ more than six-months, anti-HBe+ and HBeAg-, and undetectable serum HBV DNA level with perpetually normal aminotransferases activity. Only patients with compensated liver disease have been selected. HCs were defined as participants with no clinical signs or symptoms of the disease. Criteria for exclusion were tuberculosis, pregnancy, HCV and HIV infection, renal failure, and diabetes mellitus.

### Clinical Data Collection

The aspartate aminotransferase-to-platelet ratio index (APRI) score (4) was used to assess cirrhosis and fibrosis in participants with CHB. To do so, aspartate transaminase (AST) and platelet (PLT) levels were measured in the patients. Calculation formula was as follows: APRI score = AST/ULN × 100/PLT (10^9^/L). ULN is the upper limit of normal AST in the laboratory during the time of this study (4). When the APRI score is over 2, a patient might suffer from hepatic cirrhosis.

### Cell Preparation and Activation

Isolation of peripheral blood mononuclear cells (PBMCs) was conducted on the heparinized blood from patients with CHB-IA, HCs, and patients with CHB-IC using Ficoll-Hypaque density gradients (Sigma-Aldrich, United States). After isolation, cells were grown in an RPMI-1640 medium from Gibco (NY, United States) containing 10% fetal bovine serum at 37°C in 5% CO_2_ atmosphere with appropriate humidity. Monocyte-depleted PBMCs were isolated from the supernatant of PBMCs overnight.

Stimulation of PBMCs and monocyte-depleted PBMCs was performed with LPS (10 μg/ml; Sigma, United States), IL-12 (100 U/ml; BioLegend, United States), HBsAg protein (1.68 mg/ml; Fitzgerald, United States), HBcAg protein (1.8 mg/ml; Fitzgerald), or HBsAg + HBcAg protein mixture (.05 mg/ml) for 24 h. In blocking experiments, IgG control or functional anti-human TIGIT antibody (5 μg/ml) was added and incubated for 24 h. Recombinant Chimera CD155 Fc protein (.5 μg/ml), recombinant human TIGIT Fc Chimera protein (5 μg/ml), or IgG control was included in the culture medium to activate the TIGIT pathway. To detect the intracellular production of IFN-γ, monensin (1 μM) was added to the cultures for the last 6 h of incubation. The cells were collected after stimulation and examined by flow cytometry. The IgG control and monensin were obtained from eBioScience (San Diego, CA, United States), and the recombinant Chimera CD155 Fc protein, recombinant human TIGIT Fc Chimera protein, and IgG control were obtained from R&D Systems, United States.

### Flow Cytometry Analysis

Staining of cell surface was conducted on the collected cells, and monoclonal antibodies against TIGIT, CD3, CD56, CD69, CD25, and CD107a (eBioscience Co., San Diego, CA, United States) were added to the cell suspensions. As negative controls, isotype controls having irrelevant specificities were used. Incubation of these suspensions was done on ice for 30 min. To analyze intracellular IFN-γ, fixing and permeabilization of cells were conducted with Fixation and Permeabilization Buffer from BD Biosciences (San Jose, CA, United States). Then, the monoclonal antibody was added against IFN-γ (eBioscience Co., San Diego, CA, United States) and kept in the dark for 30 min. After washing out the samples, pellets were re-suspended in a cold 300-μl staining buffer and analyzed with a FACS Calibur cytometer (Becton Dickinson Co., United States). Analysis of data was performed using FlowJo software version 7.6.1 (Tree Star, Inc., Ashland, OR, United States).

### CD107a Degranulation Assay

The expression of CD107a was estimated to investigate the degranulation activity of NK cells (29). PBMCs (2.5 × 105) were stimulated for 24 h with 10 g/ml LPS, and were cultured in the presence or absence of 2.5 × 104 K562 cells with anti-CD107a mAb and 2 M monensin (eBioScience, San Diego, CA, United States) during the last 6 h of incubation. Then, CD3 and CD56 mAbs were used to stain the cells, which were analyzed by flow cytometry.

### Cytotoxicity Assay

Cell cytotoxicity assays for the NK cells were carried out according to the methods described in the previous study (30). In brief, purified NK cells from PBMCs of healthy participants and patients with CHB were stimulated in the presence or absence of IL-12 (100 U/ml) for 24 h and accumulated as effector cells. For being used as target cells, labeling of K562 cells was performed with carboxyfluorescein-diacetate-succinimidyl-ester (CFSE) (Sigma-Aldrich, United States). Co-incubation of target cells and effector cells was performed at an effector-to-target (E:T) ratio of 10:1 for 6 h. Only target cells were added to control tubes and used to estimate spontaneous cell death. After washing twice, 5 μl propidium iodide (PI) (eBioscience Co., San Diego, CA, United States) was added into the suspension of cells, and the resulting sample was kept in the dark for 15 min. Then, immediate analysis of cells by flow cytometry was carried out and the target cells that were dead were presented as CFSE^high^ PI + cells.

### Apoptosis Analysis

The PBMCs were cultured with LPS (24, 48, and 72 h). After harvesting, percentages of apoptosis cells were determined through Apoptosis Detection Kit from BD Biosciences (San Jose, CA, United States) as per the provided instructions.

### Statistical Analysis

Data are presented as the mean ± Standard Error of Mean (SEM). Analysis of statistical differences among groups was done applying the Mann-Whitney U test. The relationship between two factors was assessed by Spearman's rank correlation test for non-parametric data. The statistical analyses were performed using GraphPad Prism software version 5.01 for Windows (GraphPad Software Inc., San Diego, CA, USA). A value was statistically significant at *p* < 0.05 (^*^p < 0.05, ^**^p < 0.01, and ^***^p < 0.001).

## Results

### NK Cell Phenotype and TIGIT Levels in Patients With CHB

**Figure 1:**
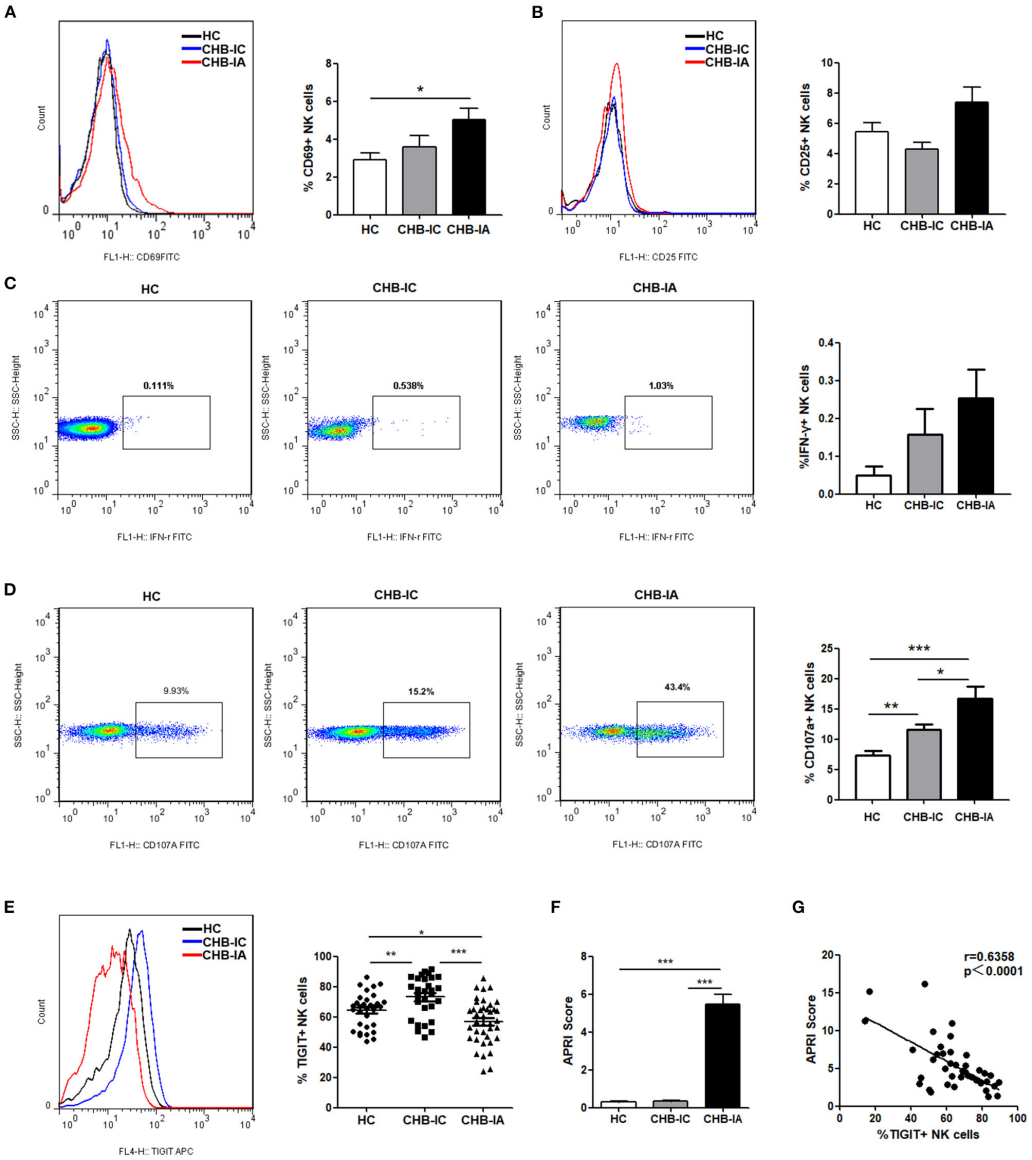
NK cells phenotype and TIGIT levels in the three groups of patients. **(A–C)** PBMCs isolated from CHB-IA, CHB-IC, and healthy individuals were analyzed by flow cytometry without any stimulate. Representative FACS histograms showing the expression of CD69 **(A)** or CD25 **(B)** on peripheral blood NK cells. The mean fluorescence intensity (MFI) of CD69 **(A)** or CD25 **(B)** is shown as mean ± SEM (n = 7~12). **(C)** Representative FACS plots showing the production of IFN-γ in peripheral blood NK cells without stimulation. The percentages of IFN-γ is also shown as mean ± SEM (n = 10 subjects per group, right). **(D)** The PBMCs isolated from the three groups were stimulated with IL-12 for 24 h with K562 as target cells. Representative FACS plots showing the expression of CD107a on peripheral. The percentages of CD107a + NK cells are shown as mean ± SEM (n = 16–23 subjects per group) (Mann-Whitney U test). **(E)** Representative FACS histograms showing the expression of TIGIT on peripheral blood NK cells. The mean fluorescence intensity (MFI) of TIGIT is also shown as mean ± SEM (n = 10 subjects per group, right). **(F)** APRI score in the three groups is shown as the mean ± SEM (n = 9 subjects per group). Data are from a single experiment representative of three. ***p 0.001 (Student's t-test). **(G)** Correlation between TIGIT and APRI scores expression on NK cells in CHB patients is shown (Spearman's rank correlation test). Each symbol represents an individual donor. Data are from a single experiment representative of three. *p < 0.05, **p < 0.01, ***p < 0.001.

The basic activation level of NK cells in patients with CHB was estimated by flow cytometry after evaluation of CD69 and CD25 expressions in the NK cells. We observed that CD69 expression on NK cells in CHB-IA Group was significantly higher than that in HC group, but showed no difference between CHB-IA and CHB-IC Group (Figure 1A). The expression of CD25 showed no difference among CHB-IA, HCs, and CHB-IC (Figure 1B). The baseline production of IFN-γ by NK cells was relatively low without stimulation, and there was no difference among the three groups (Figure 1C). CD107a expression in NK cells with K562 as target cells was increased in CHB-IA compared with that in CHB-IC and HCs (Figure 1D). Furthermore, we detected TIGIT expression in NK cells of CHB-IA, CHB-IC, and HCs, and found that the percentage of TIGIT+ NK cells decreased in CHB-IA but increased in CHB-IC compared to that in HCs (Figure 1E). We also detected the expression of TIGIT in NK cells post-stimulation with LPS or IL-12 and observed no difference (Supplementary Figure 1). To assess the relationship between liver injury and TIGIT expression, APRI score was used to assess the level of liver damage. When the APRI score is over 2, a patient might suffer from hepatic cirrhosis. The APRI score is correlated with the liver damage level. We found that the APRI score in the CHB-IA group was significantly increased compared with that in CHB-IC and HCs (Figure 1F), and that there was an inverse correlation between TIGIT expression and the APRI score in patients with CHB-IA (r = 0.6358, *p* < 0.0001) (Figure 1G).

### Association Between the Expression of TIGIT and NK Cell Phenotype in Patients With CHB-IA

**Figure 2:**
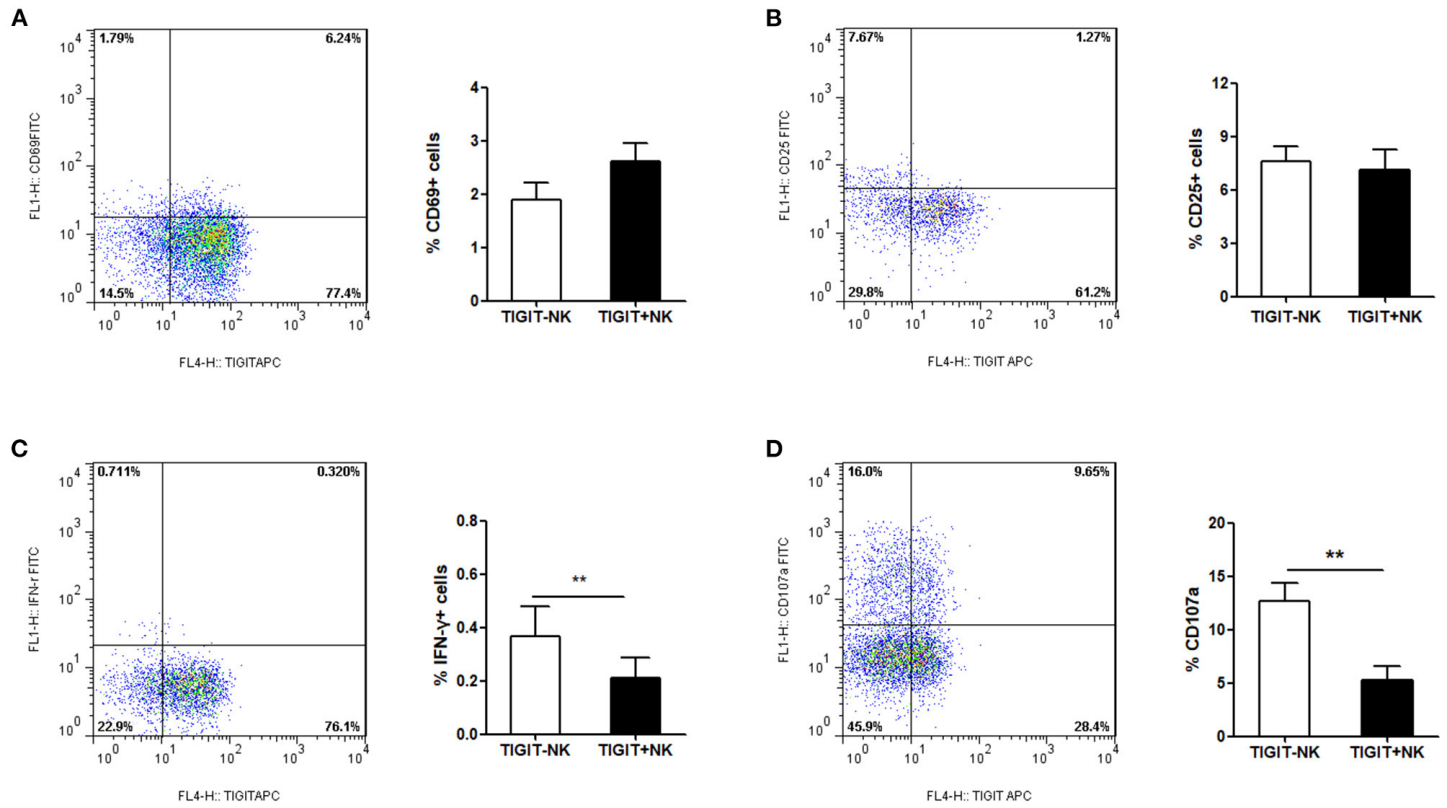
TIGIT expression and NK-cell phenotype in CHB. PBMCs isolated from healthy individuals were analyzed by flow cytometry without any stimulate. FACS plot showing the expressions of CD69 **(A)** or CD25 **(B)** on NK cells from a representative CHB-IA patient. CD69 expression on TIGIT- or TIGIT+ NK cells is shown as the mean ± SEM (n = 12 subjects per group). **(C)** FACS plot showing the expressions of IFN-γ in NK cells from a representative CHB-IA patient. Expression on TIGIT- or TIGIT+ NK cells is shown as the mean ± SEM (n = 8 subjects per group). **(D)** FACS plot showing the expressions of CD107a on NK cells from a representative CHB-IA patient. CD107a expression on TIGIT- or TIGIT+ NK cells is shown as the mean ± SEM (n = 9 subjects per group). Data are from a single experiment representative of three. *p < 0.05, **p < 0.01, ***p < 0.001.

In the next step, we tried to determine the association between the expression of TIGIT and the phenotype of NK cells in the patients with CHB-IA. We found that the percentage of CD69+ and CD25+ cells among the TIGIT-NK cells did not differ significantly from the TIGIT+NK cells in the patients with CHB-IA (Figures 2A,B). However, the TIGIT-NK cells had higher baseline IFN-γ production than the TIGIT+NK cells with no stimulation (Figure 2C). We also observed that the TIGIT-NK cells possessed significantly higher background CD107a expression than the TIGIT+NK cells (Figure 2D).

### TIGIT Expression and NK Cell Function Are Inversely Related

**Figure 3:**
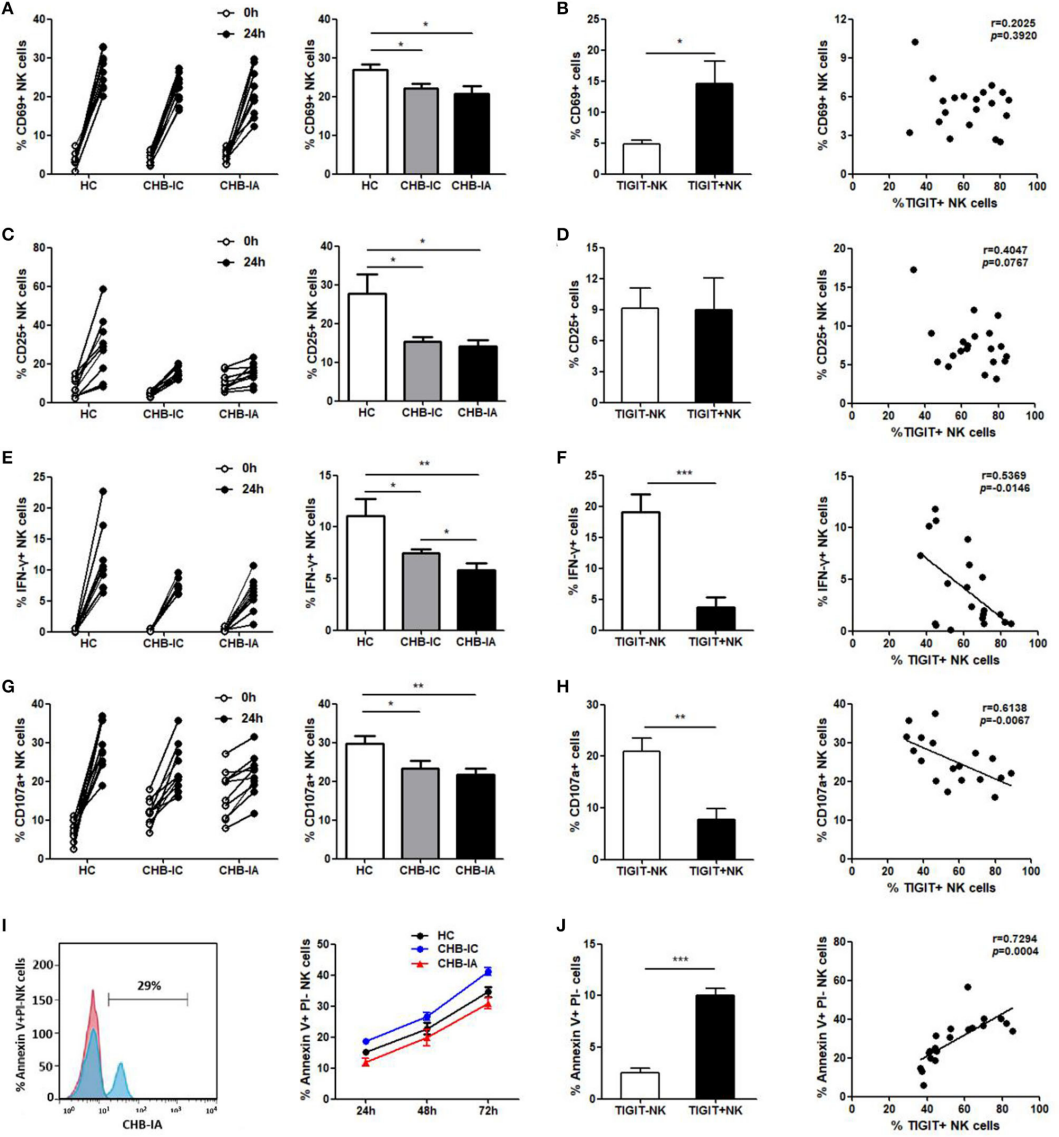
Correlations of TIGIT expression with the function of NK in different groups of the study. PBMCs isolated from CHB-IA, CHB-IC and healthy individuals were stimulated with LPS **(A–D)** or IL-12 **(E, F)** for 24 h. **(A,C)** The percentages of CD69+ **(A)** or CD25+ **(C)** NK cells before and after stimulation with LPS in the three groups were evaluated by flow cytometry. Data are expressed as the mean ± SEM (n = 8–15 subjects per group) and are from a single experiment representative of three. *p < 0.05 (Mann-Whitney U test). **(B,D)** The percentages of LPS-stimulated CD69 **(B)** or CD25 **(D)** on TIGIT- or TIGIT+ NK cells was measured by flow cytometry. Correlation between TIGIT expression and percentages of LPS-stimulated CD69 **(B)** or CD25 **(D)** on NK cells is also shown (Spearman's rank). **(E)** The percentages of IFN-γ in NK cells before and after stimulation with IL-12 in the three groups were evaluated by flow cytometry. Data are expressed as the mean ± SEM (n = 10). **(F)** The percentages of IL-12-stimulated IFN-γ in TIGIT- or TIGIT+ NK cells was measured by flow cytometry. Data are shown as the mean ± SEM (n = 10). Correlation between TIGIT expression and percentages of IL-12-stimulated IFN-γ in NK cells is also shown (Spearman's rank). **(G,H)** PBMCs were stimulated with IL-12 in the presence of K562 cells for 24 h. **(G)** The percentages of CD107a+ NK cells before and after stimulation with IL-12 in the three groups were evaluated by flow cytometry. Data are expressed as the mean ± SEM (n = 8–10 subjects per group). **(H)** The percentages of CD107a on TIGIT- or TIGIT+ NK cells was measured by flow cytometry. Correlation between TIGIT and LPS-stimulated CD107a expression on NK cells (Spearman's rank correlation test). **(I,J)** PBMCs isolated from CHB-IA,CHB-IC and healthy individuals were stimulated with LPS for 24 h, 48h and 72h. **(I)** FACS histograms showing the apoptosis of NK cells from a CHB-IA patient. The apoptosis of (Annexin V+PI-) NK cellsin CHB-IA groups was analyzed at different time points and shown as the mean ± SD (n =5 subjects per group). **(J)** The percentages of Annexin V+PI- on TIGIT- or TIGIT+ NK cells was measured by flow cytometry. Data are shown as the mean ± SEM (n = 5 subjects per group). Data are from a single experiment representative of three. *p < 0.05, **p < 0.01, ***p < 0.001.

We further examined the possible association between expression of TIGIT and NK cell activity. We found lower activation potential of NK cells in CHB-IA, so were degranulation and IFN-γ production ability. After stimulation with LPS, CD69 and CD25 levels in NK cells were lower in the patients with CHB-IA than in the HCs (Figures 3A,C). The TIGIT+ NK cells had higher CD69 expression than the TIGIT-NK cells, but no correlation between the expression of TIGIT and CD69 (CD25) was observed (Figures 3B,D). Then, we detected the capability of producing IFN-γ by the NK cells by stimulation with IL-12. Our data showed that IFN-γ production by the NK cells in CHB-IA was lower than in HCs and CHB-IC, and that it was inversely correlated with TIGIT expression (Figures 3E,F). We also found that the expression of CD107a in NK cells after K562 cell stimulation with LPS as target cells was relatively low in CHB-IA and inversely correlated with TIGIT expression (Figures 3G,H). Additionally, we assessed the apoptosis of NK cells and found that the apoptosis level of NK cells in patients with CHB-IA was remarkably less than that in HCs and CHB-IC at different time points by LPS stimulation (Figure 3I). The TIGIT+NK cells exhibited higher apoptosis than the TIGIT-NK cells, and the level of TIGIT was associated with the level of NK cell apoptosis in patients with CHB-IA (Figure 3J). Therefore, the expression of TIGIT impaired the function but promoted NK cell apoptosis in the patients with CHB-IA.

### TIGIT Pathway Blockade Enhances the Function of NK Cells in Patients With CHB-IA

**Figure 4:**
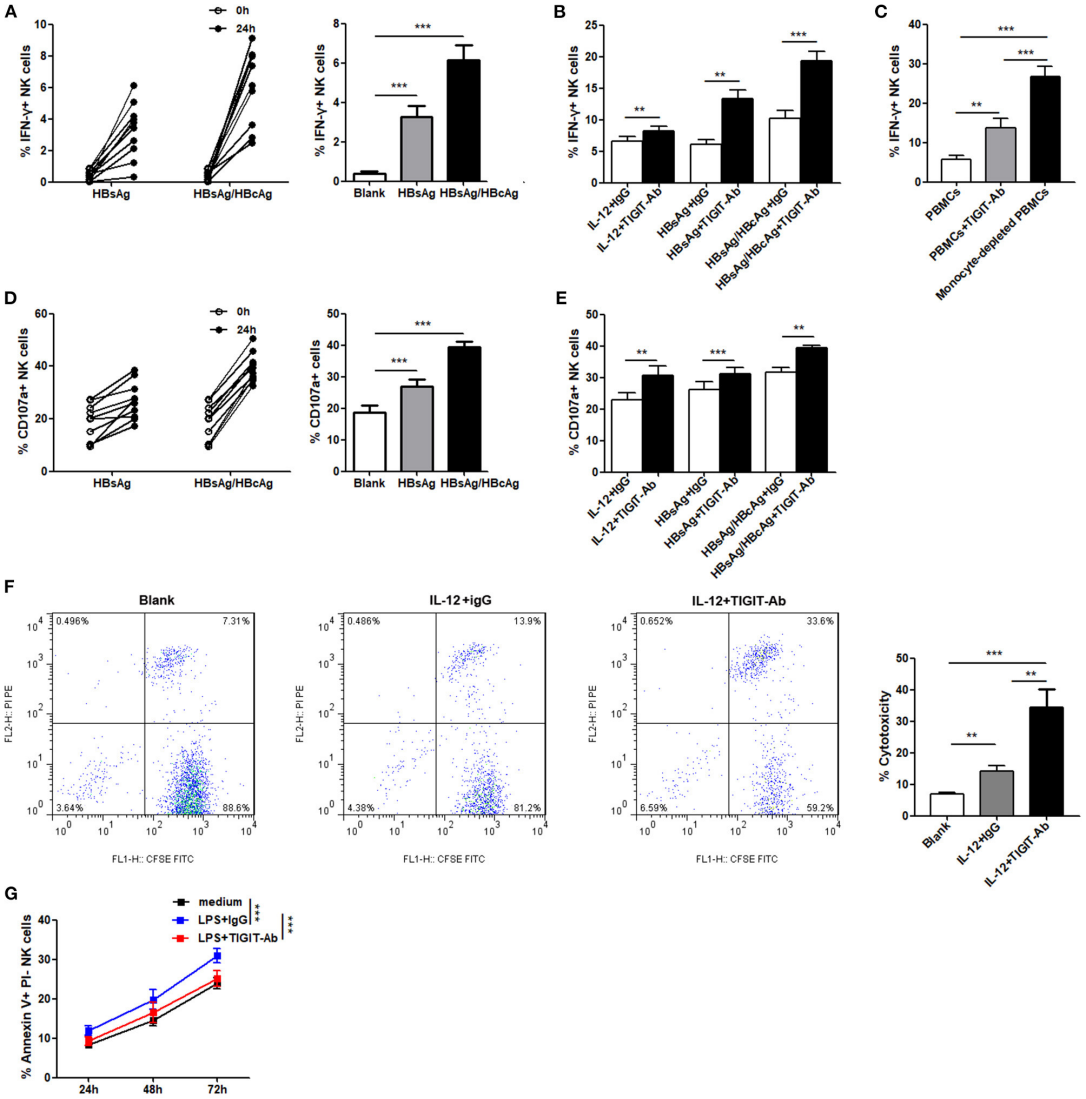
Blockade of the TIGIT pathway affects NK-cell function in CHB-IA. **(A,B,D–F)** PBMCs isolated from CHB-IA were stimulated with IL-12 or specific stimulants (recombinant HBsAg protein or recombinant HBsAg and HBcAg protein mixture) in the **(A,D)** absence or **(B,E)** presence of TIGIT-Ab protein or IgG control for 24 h. The percentages of **(A,B)** IFN-γ and **(D,E)** CD107a in NK cells before and after stimulation were evaluated by flow cytometry (n = 10). **(C)** PBMCs or monocyte-depleted PBMCs were stimulated with IL-12 for 24 h. Representative FACS dot plots showing IFN-γ expression in NK cells from PBMCs or monocyte-depleted PBMCs (n = 10). **(F)** Purified NK cells stimulated with or without IL-12 in the absence or presence of TIGIT-Ab for 24 h were collected as effector cells. K562 cells labeled with CFSE were used as target cells. E:T ratios was 1:10 for 6 h. Representative FACS plots showing cytotoxicity of NK cells. The cytotoxicity of NK cells in CHB-IA was analyzed and shown as the mean ± SD (n = 5 subjects per group). **(G)** PBMCs isolated from CHB-IA were stimulated with LPS in the absence or presence of TIGIT-Ab for 24, 48, and 72h. The percentages of Annexin V+PI- NK cells was measured by flow cytometry. Data are shown as the mean ± SEM (n = 5). Data are from a single experiment representative of three. *p < 0.05, **p < 0.01, ***p < 0.001.

We assessed whether TIGIT pathway blockade would affect the functions of NK cells in patients with CHB-IA. First, we used specific stimulants (HBsAg and HBsAg + HBcAg) and observed high IFN-γ production by the NK cells, as shown in Figure 4A. The anti-TIGIT antibody-mediated blockade of the TIGIT signaling pathway significantly enhanced the production of IFN-γ by stimulation of nonspecific and specific stimulants (Figure 4B). It has been reported that the ligand of TIGIT, CD155, was expressed in monocyte and dendritic cells. To examine the effect of TIGIT pathway blockade on the NK cells, monocyte-depleted PBMCs were used for assessment. We observed significantly enhanced IFN-γ production in the NK cells tested from PBMCs depleted of monocyte, and the effect was higher than functional blocking antibody (Figure 4C). We also observed similar results in the degranulation capacity of NK cells after TIGIT signaling pathway blockade. Specific stimulants could also induce the degranulation of NK cells (Figure 4D), and CD107a expression was significantly increased when the TIGIT pathway was blocked (Figure 4E). The cytotoxicity of NK cells was also detected, and obvious increase in cytotoxicity was observed after blocking the TIGIT pathway (Figure 4F). Moreover, the apoptosis level of NK cells was decreased by blocking the TIGIT pathway (Figure 4G). The results confirmed that the TIGIT pathway negatively regulated NK cell function and might act as a protection factor in liver damage during CHB infection.

### Activation of TIGIT Pathway Downregulates the Activity of NK Cells in Patients With CHB-IA

**Figure 5:**
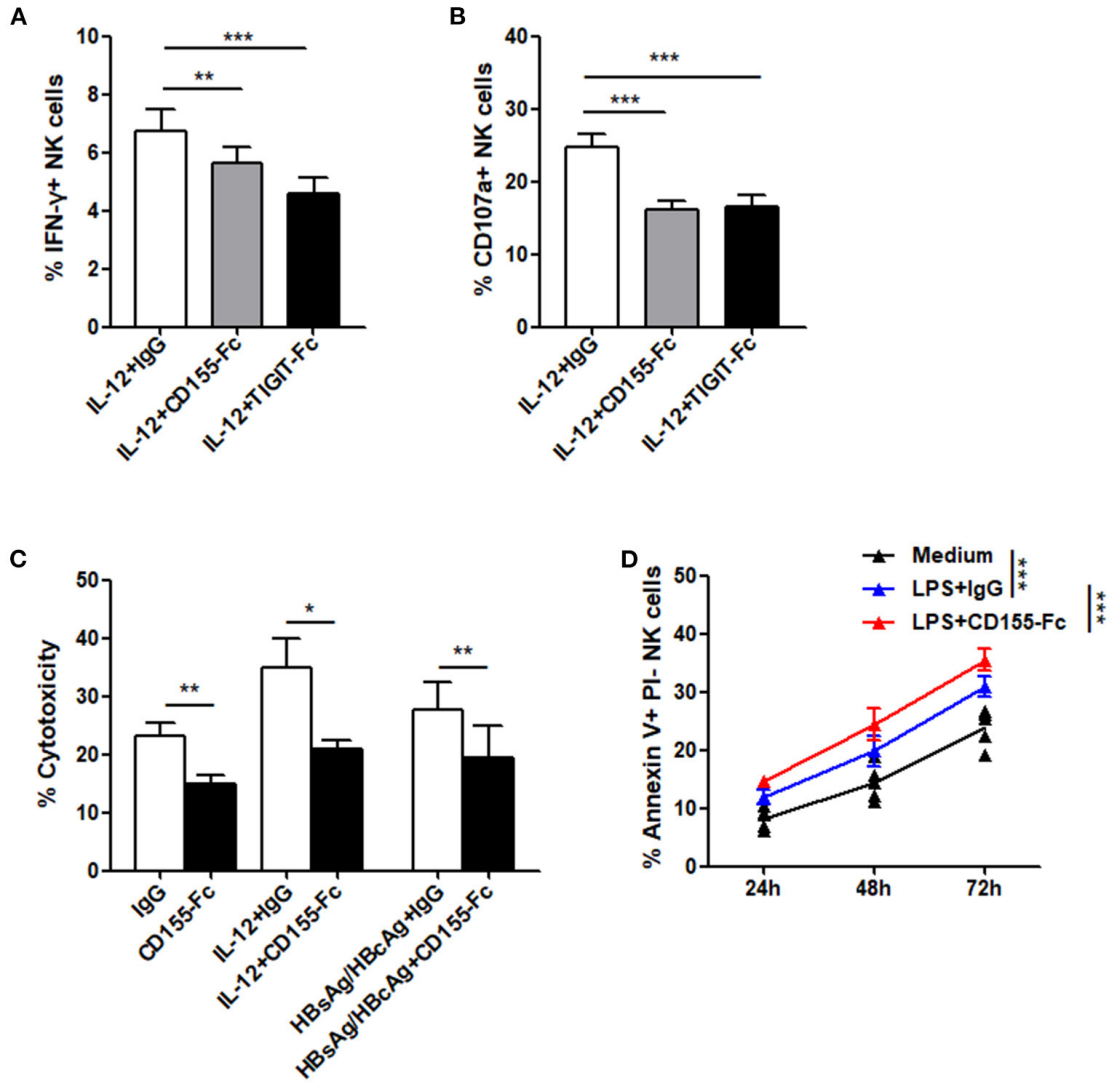
**(A–C)** PBMCs isolated from CHB-IA were stimulated with IL-12 or specific stimulants (recombinant HBsAg protein or recombinant HBsAg and HBcAg protein mixture) in the **(A,D)** absence or **(B,C)** presence of CD155Fc or TIGIT Fc protein or IgG control for 24 h. The percentages of **(A)** IFN-γ and **(B)** CD107a in NK cells before and after stimulation were evaluated by flow cytometry. **(C)** K562 cells labeled with CFSE were used as target cells. E:T ratios was 1:10 for 6 h. Representative FACS plots showing cytotoxicity of NK cells. The cytotoxicity of NK cells inCHB-IA was analyzed and shown as the mean ± SD (n = 5). **(D)** PBMCs isolated from CHB-IA were stimulated with LPS in the absence or presence of CD155Fc or IgG control or 24, 48, and 72h. The percentages of Annexin V+PI- NK cells was measured by flow cytometry. Data are from a single experiment representative of three. *p < 0.05, **p < 0.01, ***p < 0.001.

In addition, we evaluated the effect of recombinant human chimera TIGIT Fc protein and CD155 Fc protein on the function of NK cells in patients with CHB-IA by activating the TIGIT pathway. Our data reveal that IFN-γ production and CD107a expression in the NK cells following IL-12 stimulation were all significantly decreased when inducted with TIGIT Fc or CD155 Fc protein (Figures 5A,B). IL-12 and specific HBsAg + HBcAg mixture were used to activate NK cells, and cytotoxicity was also decreased after activation of the TIGIT pathway (Figure 5C). The apoptosis level of NK cells significantly increased when CD155 Fc protein was used to activate the TIGIT pathway (Figure 5D). These results indicate that TIGIT pathway activation might downregulate cytokine secretion and cytotoxicity while increasing the apoptosis of NK cells in patients with CHB-IA.

## Discussion

This study characterizes the immune activity of TIGIT in NK cells in chronic HBV infection in different stages, demonstrating that the TIGIT negatively regulates NK cells activity in chronic HBV infection, and may protect patients from severe liver injury. Consistent with the findings of earlier studies on NK cells in chronic HBV infection (9, 10), our findings showed that NK cells in patients with CHB-IA are over-activated with increased CD69 expression in NK cells and cytotoxic capacity compared to CHB-IC and HCs, but that the potential abilities of activation, IFN-γ production, and degranulation are much lower, which were the same as the previous study (31). As an active process, chronic HBV infection reflects the interaction between replication of HBV and response of the host immune system. Over-activation of NK cells in patients with CHB-IA can cause severe immunopathology of infected liver, especially increased cytotoxic activity (10). On the other hand, the IFN-γ derived from NK cells exhibits its anti-fibrotic properties by cell cycle arrest, killing activated HSCs, and HSC apoptotic induction (6, 32). In this study, the NK cells derived from the patients with CHB in the IA stage, compared with the individuals in the HC group, showed higher basic cytotoxicity while producing less or normal level of IFN-γ. Increased cytotoxicity of the NK cells can cause liver damage, while the reduced capacity of NK cells for IFN-γ production possibly decreases their antiviral activity and anti-fibrotic ability, which may aggravate liver damage, and the combination of enhanced cytotoxicity and reduced production of IFN-γ may result in liver necro-inflammation and fibrogenesis. Why IFN-γ production was lower than expected? A previous study has shown that NK cells in the liver of patients with CHB produced different amounts of IFN-γ under different stimulations (10), when higher upon PMA/ionomycin and lower upon P815/anti-NCR stimulation in comparison with peripheral NK cells. And in Jang-June Park (33) study, circulating HBeAg was shown to mediated HBV persists with virus-specific and global T-cell dysfunction. Inspired by this, we use HBsAg protein alone or combine with HBcAg protein as specific stimulants, and observed obvious changes in IFN-γ secretion and degranulation than those by using IL12. The results were similar to the previous study in which the plasmid DNA of HBV promoted the activation of NK cells in addition to cytotoxicity and production of IFN-γ in the liver (34). However, the trends did not change. Although we didn't further investigate why NK cells from IA patients produce more CD107a but not IFN-γ, but as showed in a previous study (15), in which the production of higher levels of IL-12, IL-15 and IL-18 might enhance NK activation and polarization to cytolytic activity *in vitro* in the livers of IA patients. This may be an explanation, but still need further more study. This finding supports the finding of Zhang et al. who reported the hypercytolytic activity of hepatic NK cells is correlated with the level of liver injury in patients with CHB (10). However, further studies should be conducted to shed more light on this effect.

Our group has previously assessed the expression levels of TIGIT in different types of lymphocytes in healthy individuals and found greatest expression levels of TIGIT in NK cells (35), and along with the activity of NK cells in acute infection of HBV, we supposed that TIGIT in NK cells may regulate the function of NK cells. In this study, we observed overexpression of TIGIT in NK cells in the patients with CHB-IC, but TIGIT level was much lower in NK cells of CHB-IA than the healthy individuals. This finding supports the findings of previous studies (32), in which peripheral and hepatic NK cells exhibited enhanced expressions of activation markers and receptors but reduced expression of inhibitory receptors in patients with CHB in comparison to health controls. TIGIT overexpression in IC patients might correlated with less liver damage, while lower expression in IA patients was related with sever liver injury. Liver injury was shown by APRI scores, which can evaluate and stage liver fibrosis with lower cost than FibroScan and biopsy (4). Then, we observed that the expression of TIGIT in NK cells in patients with CHB and HCs shows no change under stimulation, which seems to be stable *in vitro*, according to our previous study, in which TIGIT expression in NK cells did not respond to LPS or IL-12 stimulation (35). These results may suggest that the different clinical outcomes might be due to the different basic levels. Moreover, TIGIT level was correlated inversely with secretion of IFN-γ and NK cell degranulation, so the TIGIT-negative NK cells have greater ability for IFN-γ secretion and degranulation. These data suggested that TIGIT might be a protected marker expressed by NK cells, which prevented severe liver injury caused by HBV infection.

We examined the association between the TIGIT pathway and NK cell functional changes in patients with chronic HBV. To do so, we used a functional anti-TIGIT antibody and another recombinant TIGIT Fc chimera protein. As a non-agonistic antibody, the anti-TIGIT antibody can be used to block the TIGIT pathway (35). We found that the anti-TIGIT antibody in this study enhanced IFN-γ secretion by NK cells and cytotoxicity function but decreased NK cell apoptosis, which might obstruct liver regeneration (25) and aggravate liver damage (24). However, our study showed an opposite role for the TIGIT Fc chimera protein in a manner that its activation resulted in decreased IFN-γ secretion and cytotoxicity function but increased apoptosis of NK cells. Our finding was consistent with the finding of Chen et al. who reported that TIGIT negatively regulates inflammation by altering a macrophage phenotype (36).

The poliovirus receptor (PVR), a nectin-like family-member, is the TIGIT ligand (PVR, CD155). It is expressed in extremely low amounts in most of adult organs but is expressed in high amounts in dendritic cells (DCs), regenerating liver tissues, some tumor cells, and endothelial cells (37). T cell responses were inversely inhibited by TIGIT by modulation of cytokine profiles of mature DCs through binding with PVR (38). Then, using the CD155 Fc chimera protein, we observed that the production of IFN-γ ability and degranulation role of NK cells in patients with CHB-IA reduced, while the apoptosis level of NK cells increased. On the contrary, we removed monocytes from PBMCs to investigate the influence of PVR, then we observed that the production of IFN-γ in NK cells of patients with CHB-IA increased significantly after IL-12 stimulation, even more than blockade by anti-TIGIT antibodies, which indicates that the inhibition of the TIGIT pathway can only partly be blocked by anti-TIGIT antibodies, and that the ligand PVR (CD155) could play a more important role. TIGTI shares the ligand PVR (CD155) with CD226, which is a costimulatory molecule, but TIGIT attaches to CD155 with greater affinity than with CD226 (39–41). TIGIT pathway was only partly activated by CD155-Fc, which led to lower activation of TIGIT/PVR than by TIGIT Fc chimera protein. This might explain our finding that the suppression of IFN-γ production in NK cells by CD155-Fc was not as severe as that by TIGIT Fc. The TIGIT-PVR interaction creates a bidirectional signaling pathway, which is indicated in negatively regulated immune responses.

Some studies have analyzed hepatic NK cells and demonstrated that higher levels of activation functions and markers are exhibited by hepatic NK cells than in IA patients' peripheral NK cells, and a positive correlation of hyperactivity with liver damage in IA patients (10). In this study, we only used peripheral NK cells, not hepatic NK cells. If we could investigate the hepatic NK cells and relevant cells, including Treg cells, CD4+T cells and CD8+ cells, as well as more detail crosstalk between NK cells and other hepatocytes, it would be more persuasive. In addition, the TIGIT Fc chimera protein plays an activation role in the TIGIT/PVR pathway with enhanced secretion of IL-10 (36). In addition, IL-10 correlated with the status of HBeAg, liver disease progression, and virus replication (42, 43). In patients with positive HBeAg, higher IL-10 and IL-12 serum levels correlate with spontaneous and early seroconversion of HBeAg (44), which may be correlated with TIGIT level. In future study, we can divide patients into more detailed groups according to HBeAg status and different TIGTI levels, and observe outcomes, which may provide a clue to roughly predict the outcome of chronic HBV by TIGIT level.

## Conclusion

In conclusion, the findings of our study have revealed new evidence that TIGIT plays a protective part in chronic HBV infection. The expression of TIGIT in NK cells of patients with immune active chronic hepatitis B is lower than normal, and the level of TIGIT correlated inversely with NK-cell function in chronic HBV. This mechanism of negative regulation may represent a potential treatment target in the damage to liver by inflammatory diseases induced by acute HBV infection.

## Data Availability Statement

The original contributions presented in the study are included in the article/Supplementary Material, further inquiries can be directed to the corresponding author/s.

## Ethics Statement

Experimental procedures of this study were approved by the local Ethics Committee of Tongji Hospital, Tongji Medical College, Huazhong University of Science and Technology, Wuhan, China (Ethics code: TJ-IRB20210225) which were in accordance with the ethical standards and regulations of human studies of the Helsinki declaration (2014). Each participant provided written informed consent and was required to fill a questionnaire regarding his or her medical history and associated treatments. The patients/participants provided their written informed consent to participate in this study.

## Author Contributions

JW is the major contributor to this manuscript and wrote the first version of the manuscript. ZS conducted the analytical part and HH finalized the manuscript. JW, HH, LM, FW, JY, YL, and QL performed the experiments and analyzed the data. HH and ZS conceived and coordinated the study and critically evaluated the data. All authors read and approved the final version of the manuscript.

## Funding

This study was supported by the Infectious Diseases Control Project from Ministry of Health of China (2016ZX10004207-004) and National Natural Science Foundation of China (81401639).

## Conflict of Interest

The authors declare that the research was conducted in the absence of any commercial or financial relationships that could be construed as a potential conflict of interest.

## Publisher's Note

All claims expressed in this article are solely those of the authors and do not necessarily represent those of their affiliated organizations, or those of the publisher, the editors and the reviewers. Any product that may be evaluated in this article, or claim that may be made by its manufacturer, is not guaranteed or endorsed by the publisher.

## Supplementary Material

Supplementary Figure 1 Expression of TIGIT in NK cells post-stimulation with LPS or IL-12 in the studied groups.

## Abbreviations

ALT: alanine aminotransferase
IA: immune-active
CHB: chronic hepatitis B
HBV: hepatitis B virus
HCs: healthy controls
IC: immune-control
HCC: hepatocellular carcinoma
HBsAg: hepatitis B surface antigen
HBeAb: hepatitis B e antibody
NK: natural killer
HBeAg: hepatitis B e antigen
IFN-γ: interferon gamma
HBcAg: hepatitis c surface antigen
IL: interleukin
mAbs: monoclonal antibodies
TIGIT: T-cell immunoglobulin and ITIM domain
PBMCs: peripheral blood mononuclear cells
CFSE: carboxyfluorescein-diacetate-succinimidyl-ester
APRI: aspartate aminotransferase-to-platelet ratio index
LPS: lipopolysaccharide.
