# Supplementary figures and images for: TIGIT Signaling Pathway Regulates Natural Killer Cell Function in Chronic Hepatitis B Virus Infection
Source: Front Med (Lausanne). 2022 Feb 21;8:816474. doi: 10.3389/fmed.2021.816474 (PMC8898961; doi:10.3389/fmed.2021.816474)

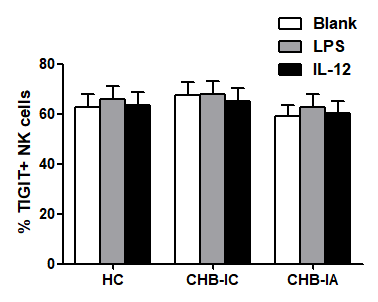

Supplement: Supplementary Figure 1 — Expression of TIGIT in NK cells post-stimulation with LPS or IL-12 in the studied groups. [file Figure_1.TIF]
